# Ameliorating the Fibrotic Remodeling of the Heart through Direct Cardiac Reprogramming

**DOI:** 10.3390/cells8070679

**Published:** 2019-07-04

**Authors:** Emre Bektik, Ji-dong Fu

**Affiliations:** Department of Medicine, Heart and Vascular Research Center, The MetroHealth System, Case Western Reserve University, Cleveland, OH 44106, USA

**Keywords:** cardiac remodeling, cardiac fibroblasts, direct cardiac reprogramming, heart regeneration, myocardial infarction

## Abstract

Coronary artery disease is the most common form of cardiovascular diseases, resulting in the loss of cardiomyocytes (CM) at the site of ischemic injury. To compensate for the loss of CMs, cardiac fibroblasts quickly respond to injury and initiate cardiac remodeling in an injured heart. In the remodeling process, cardiac fibroblasts proliferate and differentiate into myofibroblasts, which secrete extracellular matrix to support the intact structure of the heart, and eventually differentiate into matrifibrocytes to form chronic scar tissue. Discovery of direct cardiac reprogramming offers a promising therapeutic strategy to prevent/attenuate this pathologic remodeling and replace the cardiac fibrotic scar with myocardium *in situ*. Since the first discovery in 2010, many progresses have been made to improve the efficiency and efficacy of reprogramming by understanding the mechanisms and signaling pathways that are activated during direct cardiac reprogramming. Here, we overview the development and recent progresses of direct cardiac reprogramming and discuss future directions in order to translate this promising technology into an effective therapeutic paradigm to reverse cardiac pathological remodeling in an injured heart.

## 1. Introduction

Mortality by cardiovascular diseases accounts for 31.5% of all deaths worldwide [1]. The most common form of heart disease is coronary artery disease where functional cardiomyocytes (CMs) die out in the ischemic area of the heart and are replaced by a fibrotic scar, which leads to the dysfunction of the heart and eventually heart failure. Since adult CMs possess very limited to no self-regenerative capability, heart transplantation is still the final solution for patients with end-stage heart failure, however, this remains a limited option for most patients due to the shortage of donor organs. Therefore, developing new strategies of cellular therapies offers more accessible options for a broader group of coronary heart patients and prevents a diseased heart from end-stage failure. Currently, there are five major cellular therapy strategies that have been actively investigated in the past few decades for cardiac regenerative medicine: (1) Transplantation of autologous adult stem cells [2,3,4,5], (2) transplantation of embryonic stem cell (ESC)- or induced pluripotent stem cell (iPSC)-derived CMs [6,7,8], (3) activation of endogenous progenitors [9,10,11,12], (4) cell-cycle reentry of adult CMs [13,14,15,16], and (5) direct cell fate reprogramming [17,18,19,20,21]. Each strategy has shown promising benefits but are also facing different technical challenges [22,23]. Many research progresses of direct cardiac reprogramming have been discussed in recently review articles [24,25,26]; in this article, we reviewed the newest discoveries of direct cardiac reprogramming, including cell cycle regulation, chemokine signaling, inflammatory immune signaling, and single-cell RNA-seq findings during reprogramming of induced cardiomyocyte-like cells (iCM), and incorporated a new understanding of matrifibrocytes during cardiac fibrotic remodeling to discuss future directions of translating this promising technology into clinical applications.

## 2. Pathological Remodeling of the Heart

**Figure 1:**
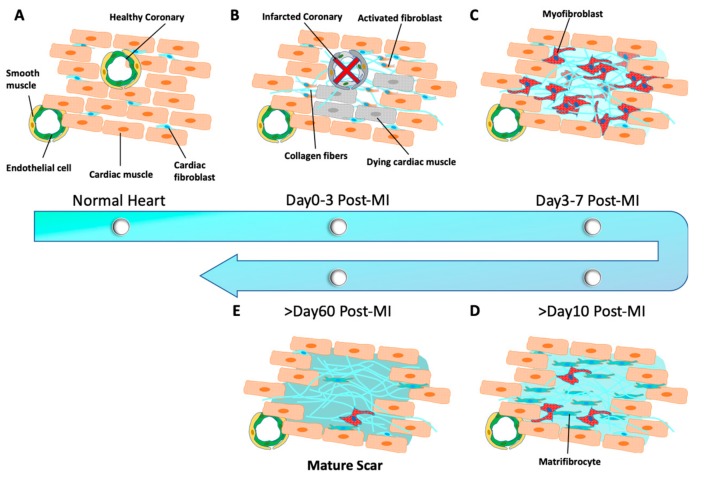
Schematic representation of cardiac remodeling progression in post-infarction mouse hearts. (**A**) Cardiac fibroblasts are normally quiescent in the heart. (**B**) Cardiac fibroblasts are activated and quickly reenter the cell cycle after myocardium infarction (MI). (**C**) Cardiac fibroblasts differentiated into myofibroblasts. (**D**) Matrifibrocytes are further differentiated from myofibroblasts. (**E**) A mature scar is formed to mechanically support and protect the heart.

During the development of coronary artery diseases, such as myocardial infarction (MI), the heart tissue undergoes a pathological remodeling where necrotic myocardium is replaced by non-myocyte cells to preserve functional and structural integrity of the heart. Remodeling is characterized by excessive accumulation of collagen-based extracellular matrix (ECM) majorly from resident cardiac fibroblasts [27]. Previous studies [28,29] have suggested that fibroblasts constitute the majority of non-myocytes in the heart, which was considered recently to be overestimated in the adult murine and healthy human heart [30]. In the normal healthy heart (Figure 1A), cardiac fibroblasts remain quiescent and play an essential role in the maintenance of mechanical, structural, and electrophysiological functions of the heart [31,32]. However, in an infarcted myocardium, quiescent fibroblasts are activated to secrete various pro-inflammatory factors [33] and quickly re-enter the cell cycle (Figure 1B). Following a massive proliferation, cardiac fibroblasts differentiate into contractile myofibroblasts that are characterized by the expression of α-smooth muscle actin (αSMA) within a week of a MI [33,34,35] (Figure 1C). Toward the end of remodeling, myofibroblasts exit the cell-cycle and provide a stronger contractile support to the damaged heart by secreting much more ECM than fibroblasts [36,37,38]. In most tissues, myofibroblasts undergo apoptosis when the scar is fully matured and the healing process is completed [39,40]. However, in the heart, a significant number of quiescent myofibroblasts persist in the formed mature scar for long time (>60 days post-MI) to mechanically support and protect the heart against any further damage [41]. Very recently, Fu et al. identified a more specialized type of cell, termed matrifibrocytes, with expression of new marker genes, such as bone-cartilage markers *Chad* and *Comp*. They found that matrifibrocytes were further differentiated from myofibroblasts and persisted in the chronic myocardial scar [42] (Figure 1D). Their lineage-tracing studies has updated our understanding of the pathological remodeling in an infarcted heart. In brief (Figure 1), quiescent cardiac fibroblasts are activated upon injury and reach a maximum proliferation rate within Day 2–4 post-MI, followed by αSMA^+^ myofibroblast differentiation and proliferation by Day 3–7 post-MI; from Day 10 post-MI, myofibroblasts lose proliferative ability and αSMA expression and then further differentiate into matrifibrocytes and finally form a mature scar within 60 days [39]. However, the role of matrifibrocytes in heart remodeling and regeneration has yet to be understood.

## 3. Development of Direct Cardiac Reprogramming

### 3.1. Direct Reprogramming of Mouse Fibroblasts into iCMs In Vitro

Since lost CMs in an injured heart are replaced by cardiac fibroblasts, it will be a promising therapy for cardiac regenerative medicine if proliferated cardiac fibroblasts can be transdifferentiated into functional CMs. Transdifferentiation was initially reported in the early 1990s whereby a single transcription factor, MyoD, was sufficient to convert fibroblasts and epithelial cells into skeletal muscle cells [43]. After decades of efforts, the discovery of iPSCs [44,45] demonstrated that, rather than a single transcription factor, a combination of several transcription factors might be required to directly convert a terminally-differentiated cell type into another. Indeed, in 2010, Ieda et al. [17] cultured cardiac fibroblasts, isolated from αMHC-GFP transgenic mice, and successfully identified a minimal combination of three transcription factors (GMT: *Gata4*, *Mef2c*, and *Tbx5*) that could directly convert cardiac fibroblasts into induced cardiomyocyte-like cells (iCMs) without undergoing an intermediate pluripotency or progenitor state. Reprogrammed αMHC-GFP^+^ iCMs expressed a group of cardiac genes, e.g., Myh6, Actc1, Actn2, etc., and showed assembled sarcomere structures. Spontaneous calcium oscillations/transients were observed in many iCMs two weeks after reprogramming induction, but action potential and cell contraction were developed only in a very small population (0.01–0.1%) of reprogrammed cells.

Soon after the first study by Ieda et al. [17], Song et al. [21] included Hand2 in the GMT cocktail (GHMT), which yielded more αMHC-GFP/cardiac Troponin-T (cTnT) double-positive iCMs in adult murine tail-tip fibroblasts and cardiac fibroblasts than GMT only. Addis et al. [46] used a different reporter system in which a genetically encoded calcium indicator GCaMP was driven by cTnT promoter. They found that Nkx2.5 could significantly enhance the efficiency of GHMT to reprogram more functional iCMs with spontaneous calcium oscillations and beating. Protze et al. [47] screened triple combinations of a group of candidate transcription factors and found that an optimal combination of Mef2c, Tbx5, and Myocd could activate expression of a broader spectrum of cardiac genes than GMT did. Hirai et al. [48] fused MyoD domain to Mef2c and found that MyoD-Mef2c fused together with three other wild-type genes (Gata4, Hand2 and Tbx5) could yield 15-fold more beating iCMs than wild-type GHMT. In contrast to the transcription-factor-based approach, Jayawardena et al. [19] investigated various combinations of cardiac enriched microRNAs (miRs) for iCM induction and found that a combination of muscle-specific miRs (miR combo: miR1, miR133, miR208, and miR409) could induce iCMs from mouse cardiac fibroblasts. Moreover, JAK inhibitor improved the reprogramming capability of the miR combo. Consistently, Muraoka et al. [49] found that miR133 suppressed Snai1, a master regulator of epithelial-to-mesenchymal transition improved GMT-mediated iCM reprogramming, including a higher yield and shortened induction time of beating iCMs. Importantly, the reprogramming trajectory is acquired within 48 h of GMT virus infection into mouse cardiac fibroblasts [50], and a sufficient expression of all reprogramming factors in individual fibroblasts is critical to overcome the initial barrier for a successful iCM reprogramming [50,51].

### 3.2. In Situ Reprogramming of iCMs in The Heart

After the success of *in vitro* iCM-reprogramming, converting resident cardiac fibroblasts into functional CMs *in situ* is critical to translating this promising approach into a practical therapeutic paradigm for cardiac regenerative medicine. The first success of *in vivo* reprogramming was reported by Qian et al. [20]. They found that a local injection of GMT retroviruses into the ischemic region of the heart, right after coronary artery ligation, could reprogram new iCMs with improved ejection-fraction of the heart and decreased scar size. They utilized multiple lineage-tracing animals, including periostin-Cre and fibroblasts-specific protein 1 (Fsp1)-Cre mice, and demonstrated that *in vivo* reprogrammed iCMs predominantly originated from resident cardiac fibroblasts. Importantly, *in vivo* reprogrammed iCMs were highly similar to native CMs with a well-formed sarcomere and functionally coupled well with endogenous CMs. Meanwhile, Song et al. [21] also showed the success of *in vivo* iCM-reprogramming with GHMT factors. Most importantly, both studies have shown that *in vivo* generated iCMs improve the ejection-fraction and decrease scar size four weeks after MI. Additional Thymosin-β4 could improve the outcome of GMT-reprogramming and further enhance the functional recovery of the infarcted heart through improved neovascularization [52]. The success of *in vivo* reprogramming has also been reproduced with a miR combo (miR-1, miR-133, miR-208, miR-499), which decreased scar size and improved heart function in the mouse MI model [19,53]. Inagawa et al. [18] found that GMT viruses majorly infected fibroblasts and other non-myocyte cells within the scar, but only 3% of infected cells expressed αMHC-GFP and formed striated sarcomeres. A single polycistronic vector of three factors (GMT) with self-cleaving peptides could increase the *in vivo*-reprogramming yield of αMHC-GFP^+^/α-Actinin^+^ cells, of which 30% showed well-formed sarcomere structures. Consistently, Wang et al. [54] studied different orders of three factors in single polycistronic vector and found that a polycistronic vector of MGT, in which Mef2c is expressed at relatively higher levels than Gata4 and Tbx5, could improve the efficiency and the quality of *in vitro*-reprogrammed iCMs. However, MGT construct only increased the number but not the quality of *in vivo*-reprogrammed iCMs [55], suggesting that the *in vivo* environment of the heart has a more significant role in iCM quality than MGT itself. Nonetheless, single-cell transcriptome analysis revalidated that Mef2c is expressed relatively higher than Gata4 and Tbx5 in successfully reprogramming iCMs [56].

### 3.3. Direct Cardiac Reprogramming of Human Fibroblasts

Another critical step in translating direct cardiac reprogramming into a therapeutic approach is to achieve reprogramming of human cells with optimized reprogramming cocktails. Several research teams, including ours, has shown that neither GMT nor GHMT were sufficient to reprogram human fibroblasts. Wada et al. [57] found that the addition of Myocd and Mesp1 to GMT (GMTMM) could convert human cardiac and dermal fibroblasts into iCMs, in which the expression of cardiac genes were increased and fibroblast genes were decreased. In our laboratory, we re-performed a screening of 21 cardiac enriched transcription factors and found that Esrrg and Mesp1 together with GMT (5F) was sufficient to induce αMHC and cTnT double-positive iCMs from human fibroblasts [58]. Moreover, addition of Myocd and ZFPM2 to 5F combination (7F) further increased the yield and improved the quality of reprogrammed iCMs. Global gene expression in iCMs shifted from fibroblasts to cardiomyocyte-like state. Calcium transients and a depolarized resting membrane potential were observed in both 5F- and 7F-reprogrammed cells; however, spontaneous contraction was not observed. Our single cell qPCR studies demonstrated that both Hand2 and miR1 could further improve the quality of 7F-reprogrammed iCMs, indicated by more activated cardiac genes and silenced fibroblast genes [59], although the yield of iCMs was not improved. Meanwhile, TGF-β and Wnt signaling inhibitors could improve the 7F-iCM reprogramming and induced more spontaneous calcium oscillations in 7F-iCMs [60]. Nam et al. [61] started from GHMT and screened additional transcription factors for human iCM reprogramming. They found that Myocd together with GHMT could activate more cTnT expression in human fibroblasts. They further included miR1 and miR133 and identified a reprogramming cocktail of six factors (Gata4, Hand2, Tbx5, Myocd, miR1, and miR133) that could directly convert human fibroblasts into iCMs. Spontaneous calcium oscillation was observed in 8-week reprogrammed iCMs and spontaneous contraction was observed in a very small portion of iCMs 11 weeks after reprogramming.

### 3.4. Direct Reprogramming to Multipotent Cardiac Progenitors

In addition to iCM reprogramming, reprogramming fibroblasts into expandable cardiac progenitor cells has also been studied. A combination of five factors (Mesp1, Tbx5, Gata4, Nkx2.5, and Baf60c) in combination with activation of canonical Wnt and JAK/STAT pathways could reprogram adult mouse cardiac, lung, and tail tip fibroblasts into expandable progenitor cells, termed induced cardiac progenitor cells (iCPCs) [62]. iCPCs display cardiac-mesoderm-restricted multipotency with the ability to be differentiated into cardiac myocytes, smooth muscle, and endothelial cells. Considering the chemically defined condition that can capture and expand CPCs *in vitro* [63], reprogramming of iCPCs provides an alternative cell source for patient-specific autologous cell transplantation therapy.

## 4. Mechanistical Understanding of Direct Cardiac Reprogramming

With the goal of translating reprogramming into a therapeutic paradigm, scientists around the world have been enthusiastically investigating the reprogramming mechanism and developing new approaches/tools to improving the efficiency of direct cardiac reprogramming.

### 4.1. Activation of Signaling Pathways During Reprogramming

**Figure 2:**
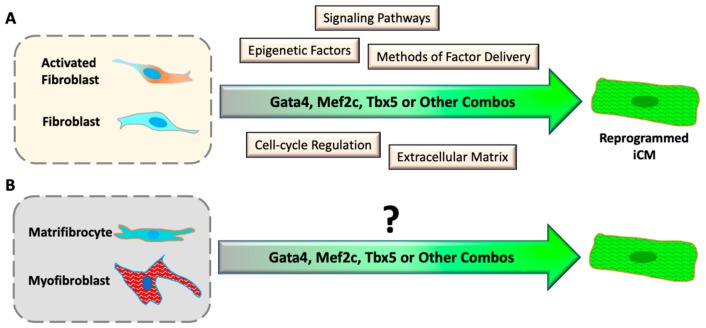
Progresses and challenges of direct cardiac reprogramming *in vitro*. (**A**) Understanding the mechanism of iCM reprogramming with cultured inactive and activated fibroblasts. (**B**) It is unknown if differentiated myofibroblasts and matrifibrocytes can be reprogrammed into iCMs.

It has been found that different signaling pathways are involved in and directly regulate iCM reprogramming (Figure 2A and Table 1). Inhibition of transforming growth factor beta (TGF-β) by SB431542 compound [64,65] could enhance GHMT-reprogramming and yield more iCMs from mouse embryonic fibroblasts (MEFs) and adult cardiac fibroblasts. Inhibition of Rho-associated kinase (ROCK) pathway increased the conversion rate of iCM-reprogramming in MEFs, tail tip fibroblasts, and cardiac fibroblasts [65], probably by the prevention of cell apoptosis that happens in some newly-reprogrammed iCMs [66]. Inhibition of Notch signaling increased the binding of Mef2c to the promoter regions of cardiac structural genes and subsequently enhanced cardiac reprogramming [67]. Furthermore, a combination of Wnt-inhibitor and TGF-β inhibitor could significantly augment GMT reprogramming and accelerate the conversion progression of beating iCMs [60]. In addition, activation of IGF1/PI3K/Akt1 signal pathway, in which mTORC1 and Foxo3a act as downstream mediators, enhanced GHMT-mediated reprogramming [68]. The reprogramming benefit of activation of p38 MAP kinase and PI3K/Akt pathways was consistently observed with a serum-free culture condition of FGF2, FGF10, and VEGF (FFV) [69]. On the other hand, suppression of inflammatory signaling has been shown to enhance direct cardiac reprogramming. For example; the zinc finger transcription factor 281 (ZNF281) enhanced cardiac reprogramming in part by repressing the inflammatory markers in adult mouse tail-tip fibroblasts [70]. Recently, inhibition of C-C chemokine signaling in MEFs or neonatal cardiac fibroblasts enhanced cardiac reprogramming efficiency, emphasizing a requirement of immune-response suppression for iCM-reprogramming progression [71]. Consistently, inhibition of inflammatory immune signaling through suppression of cyclooxygenase-2 highly improved the yield and quality of reprogrammed iCMs in neonatal and adult mouse fibroblasts [72], which was not observed in MEFs.

### 4.2. Epigenetic Barriers of Reprogramming

Direct reprogramming of somatic cells into other type of cells, including iPSCs [73,74], neurons [75,76] and CMs, requires overcoming epigenetic barriers and the opening of chromatin structures on critical genes related to specific cell-fate identity. Indeed, the enrichment of trimethylated histone H3 of lysine 4 (H3K4me3), a mark of actively-transcribed genes, was increased and trimethylated histone H3 of lysine 27 (H3K27me3), a mark of inactive genes, was decreased in cardiac gene promoter regions as early as Day 3 of cardiac reprogramming [77]. Interestingly, the most effective timing of H3K27me3 diminishment to improve iCM-reprogramming was observed from Day 1 to Day 4 after virus infection, while diminishment of H3K9me2, another mark of inactivated genes, was most effective in enhancing the reprogramming from Day 3 to Day 7 [78], suggesting that the timing of epigenetic regulation is critical for a successful direct cardiac reprogramming. Inhibition of H3K27 methyltransferases or of polycomb repressive complex 2 (PRC2) could induce cardiac gene expression in fibroblasts; while inhibition of H3K27 demethylases blocked the induction of cardiac gene expression in miR-combo-mediated reprogramming [79]. Consistently, suppression of Bmi1, one component of PRC1, enhanced open chromatin states of cardiac genes, especially of Gata4, which improved cardiac reprogramming and eliminated the need for exogenous Gata4 [80]. Nonetheless, ZNF281, a transcriptional regulator, co-occupied cardiac gene enhancers with Gata4 and promoted GHMT(+Akt1)-mediated cardiac reprogramming in adult mouse fibroblasts through synergistic activation of diverse set of cardiac genes [70]. Very recently, it was found that Mef2c orchestrates chromatin accessibility of Gata4 and Tbx5 factors by recruiting them to Mef2 binding sites [81]. On the other hand, ectopic Gata4 was shown to be enriched at low levels on additional target gene loci and co-expression with another pioneer factor, Foxa2, increased enrichment on sampling genomic sites [82].

### 4.3. Cell-Cycle Regulation During Direct Cardiac Reprogramming

Although cardiac fibroblasts are quiescent in the healthy heart [31]; fibroblasts in an injured heart or in culture are quickly activated to re-enter the cell cycle. It is known that each cell-cycle phase constitutes a chain of interconnected events with a dynamic fluctuation of epigenetic chromatin modifications, including genomic DNA methylation and histone modifications [83,84]; therefore, it is important to understand how cell cycle is regulated in reprogrammed cells during direct cardiac reprogramming. Time-lapse imaging from Day 2 to Day 4 post-GMT-infection showed directly that nearly 40% of αMHC-GFP^+^ iCMs reprogrammed from MEFs were still active in the cell cycle at early stages of reprogramming [66], but αMHC-GFP^+^ iCMs gradually exited the cell cycle along with the progression of reprogramming. Interestingly, time-lapse imaging of early-stage reprogrammed cells suggested that iCM-reprogramming was mostly initiated at late-G1- or S-phase [66], indicating that a particular phase of the cell cycle might offer a more optimal epigenetic status for reprogramming induction. Indeed, depletion of Foxa2 in human foreskin fibroblasts resulted in a decreased demethylation on Foxa2 target genes in S-phase, but not in G1-arrested cells [82], suggesting that a loosened structure of S-phase chromosomes assists transcription factors access to their targeted genes. In our study, cell-cycle synchronization at S-phase facilitated cell-cycle exit and accelerated iCM reprogramming with enhanced expression of cardiac genes [66]. Other studies have also demonstrated that cell-cycle exit is critical for the maturation of reprogrammed iCMs from MEFs and mouse cardiac fibroblasts [66,85], while overexpression of large T antigen, which endows cardiac fibroblasts a constitutive proliferation ability, inhibited reprogramming induction [56]. Recently, by single-cell analysis techniques, critical function of cell-cycle exit is validated in iCM reprogramming of human cardiac fibroblasts [86]. In summary, cell-cycle exit, which is a developmentally required process of CM maturation in mammalian hearts [87], is an important and necessary event for direct cardiac reprogramming.

### 4.4. Modification of Extracellular Matrix

Extracellular matrix (ECM) has a significant impact on the function of cardiomyocytes. Matrices that mimic mechanical stiffness of the developing heart is optimal for promoting actomyosin striation and for transmitting contraction of cardiomyocytes to the matrix; neither harder matrices, which mimic the ECM of infarcted heart, nor softer matrices are good for developing well organized sarcomeres in cardiomyocytes [88,89]. Manipulating the stiffness of culture matrix could improve the functional maturation of human iPSC-derived cardiomyocytes [90]. Indeed, ECM also plays an important role in cardiac reprogramming (Table 1). Although adjusting the substrate stiffness alone didn’t improve reprogramming yield, a micro-grooved substrate could enhance reprogramming efficiency and yielded more mature iCMs with improved quality, including the higher expression of cardiac genes and more beating cells, through an increased nuclear localization of Mkl1, a mechanosensitive transcription factor [91]. Li et al. [92] encapsulated reprogrammed fibroblasts in three-dimensional (3D) hydrogels and found that the 3D tissue bundle environment increased the expression of matrix metalloproteinases (MMPs) and enhanced direct cardiac reprogramming with increased yield and improved quality of iCMs. These findings demonstrate the importance of a proper extracellular-matrix for reprogramming, which is one of the mysterious *in vivo* environmental factors in the native heart. Although these studies partly mimic the *in vivo* environment of a scar, an actual scar exhibits different levels of stiffness [93] with different amounts of collagen deposition [94] at various time points after infarction, which may affect reprogramming quality differently. Therefore, a specific period of post-MI time window may have an optimal stiffness environment to maximize the reprogramming efficiency *in vivo*. Indeed, infarcted myocardium progressively increased stiffness within a month and the highest level of regeneration was achieved at Day 7–14 post-MI, which provided an optimal stiffness for the endothelial progenitor lineage commitment of transplanted bone-marrow mononuclear cells [93]. Therefore, future reprogramming studies should pay greater attention to the mechanical microenvironments in cell cultures and *in vivo* studies.

### 4.5. Non-Genomic Integration Methods for Direct Cardiac Reprogramming

It is a clinical concern that retroviruses/lentivirus integrate into the genome of host cells and might insert random mutations [21,95,96,97,98,99]. Therefore, non-genomic integration methods have also been investigated actively for a safer approach for iCM reprogramming. In addition to adeno and adeno-associated virus that were studied in reprogramming [98,100], Miyamoto et al. [101] utilized Sendai virus (SeV) and developed a non-integrating, polycistronic GMT vector system (SeV-GMT) that successfully reprogrammed cardiac fibroblasts into iCMs *in vitro* with higher reprogramming efficiency. *In vivo* delivery of SeV-GMT yielded a better functional recovery of the heart with ~3.5 times smaller scar size than retroviral-GMT did. Chang et al. [102] utilized cationic gold nanoparticles (AuNPs) to deliver GMT transcription factors into MEFs. AuNPs allowed integration-free transfection of reprogramming factors with no measurable cytotoxicity on cells. Fibroblasts were much more efficiently reprogrammed into iCMs and started beating with AuNP-GMT compared to regular GMT transfection. Moreover, *in vivo* delivery of AuNP-GMT in a mouse model improved heart function by measurably decreasing scar size and fibrotic area two weeks post-MI. Alternative to gene therapy, a cocktail of small-molecule chemical compounds, consisted of CHIR99021, RepSox, Forskolin, VPA, Parnate, and TTNPB (CRFVPT), had been identified to successfully reprogram MEFs and tail-tip fibroblasts into chemical-induced cardiomyocyte-like cells (CiCMs) [103]. The induction of CiCMs by CRFVPT passes through a cardiac progenitor stage with upregulated expression of Sca-1, Wt1, Flk1, Abcg2, and Mesp1 in the early stages of reprogramming, but not a pluripotent stage. Importantly, *in vivo* delivery of CRFVPT plus Rolipram into Fsp1-Cre:R26R^tdTomato^ mice successfully generated tdTomato^+^ CiCMs in the heart and reduced the formation of fibrotic scar tissues in MI hearts with functional improvement of the heart [104]. Similarly, CiCMs could be also reprogrammed from human foreskin fibroblasts by a combination of nine chemical compounds (CHIR99021, A83-01, BIX01294, AS8351, SC1, Y27632, OAC2, SU16F, and JNJ10198409) [105]. Human CiCMs also initially passed through a progenitor stage before the final conversion into functional iCMs, suggesting a different mechanism of CiCM-reprogramming from gene-mediated cardiac reprogramming. All these studies with non-integrated approaches will facilitate the translation of direct cardiac reprogramming into an efficient and safer therapy for future clinical applications.

## 5. Perspective to Translate Direct Cardiac Reprogramming

Despite the enormous progress, direct cardiac reprogramming remains an immature technology and a better mechanistic understanding of iCM reprogramming, especially *in vivo* reprogramming, is required for future clinical applications.

### 5.1. Understanding the Mystery of In Vivo Reprogramming

Compared to *in vitro* reprogramming in petri dishes, *in vivo* reprogramming generated a higher yield and better quality of iCMs in mouse heart with acute MI [18,20,21], suggesting that the *in vivo* environment of the heart after acute MI appears to overcome more cellular and epigenetic barriers and improves direct cardiac reprogramming. Upon myocardial injury, fibroblasts as well as other non-myocyte cells, e.g., macrophages, are being activated to remodel infarcted heart tissue. Cardiac resident macrophage proliferation occurs within the first week following pressure overload hypertrophy and is a requisite for the heart’s adaptive response [106]. It is still unknown how much of those activated inflammation signals contribute to the enhancement of *in vivo* iCM-reprogramming. The close interactions between various cell types in the infarct and peri-infarct zone could play a critical role in improving cardiac reprogramming. For example, cardiac resident mesenchymal stem cells (MSCs) secrete molecules that protect the heart against damage [107]; therefore, secreted factors might improve the efficiency of direct cardiac reprogramming, while reprogramming factors may also have positive effects on cardiac MSCs in terms of secreting more cardioprotective and regenerative factors. In addition, the extracellular matrix in the heart provides proper stiffness and a topographical 3-D microenvironment, which is important for the induction and maturation of reprogrammed iCMs [91,92]. We speculate that direct mechanical and/or electrical interactions between iCMs and neighbor myocytes/non-myocytes/ECM factors, combined with endogenously secreted factors, may cooperatively work to improve direct cardiac reprogramming, which should be carefully studied and well understood before its clinical application.

### 5.2. Converting Chronic Fibrotic Scar into Myocardium

**Figure 3:**
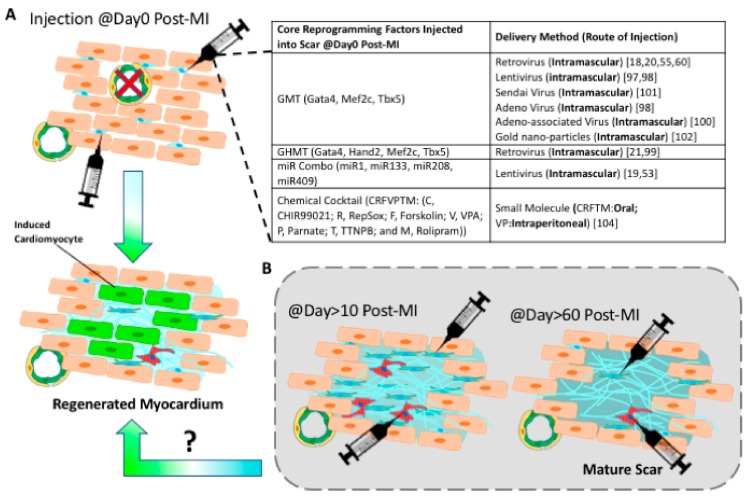
Progresses and challenges of *in vivo* reprogramming. (**A**) Cardiac reprogramming factors were all delivered right after acute myocardial infarction. The inserted table summarizes *in vivo* reprogramming studies that use various viral or chemical cocktails [18,19,20,21,53,55,60,97,98,99,100,101,102,104]. (**B)** It is unknown if a cardiac chronic scar can be reprogrammed into induced cardiac muscle tissue.

Noticeably, all published studies of *in vivo* reprogramming were performed with an acute MI model, with gene delivery right after coronary artery ligation (Figure 3A). Therefore, those freshly activated cardiac fibroblasts are the major cells targeted by different combinations of reprogramming factors. However, the majority of activated fibroblasts have been differentiated into αSMA^+^ myofibroblasts and further become αSMA^−^ matrifibrocytes in a chronic scar [42]. So far, it is unknown whether these differentiated myofibroblasts/matrifibrocytes are reprogrammable *in vitro* (Figure 2B) and *in vivo* (Figure 3B). Several studies have demonstrated that achieving a successful cardiac reprogramming *in vitro* requires healthy and non-senescent fibroblasts [108,109,110]. In addition, treatment of virus-infected fibroblasts with TGFβ, which induces myofibroblasts differentiation, inhibits cardiac reprogramming [65], indicating the difficulty of reprogramming iCMs from myofibroblasts/ matrifibrocytes. Since there is still no effective treatment for a chronic cardiac scar, it will be priceless to develop a novel approach that can reprogram myofibroblasts/matrifibrocytes into iCMs, which is the first step to eventually converting chronic cardiac scar into functional muscle tissue, and will accelerate the translation of direct cardiac reprogramming. As we discussed earlier, variations of scar stiffness and size across the infarct might be another important aspect of reprogramming the chronic scar into iCMs, which needs to be addressed in future studies.

### 5.3. Direct Cardiac Reprogramming of Human Cells

Conversion of human fibroblasts into beating functional iCMs is still very challenging and not much progress has been made since the first three publications [57,58,61], although many mechanistic understandings of cardiac reprogramming have been achieved with mouse cells (Table 1). One realistic hurdle is that there is a very limited source to obtain high-quality human cardiac fibroblasts that, as found in mouse cells, is critical for the success of direct cardiac reprogramming. It is a consistent finding that human iCM reprogramming requires more reprogramming factors and takes a longer time than mouse reprogramming [57,58,61], especially because the conversion rate of human iCMs is still very low. The most recent study by Zhou et al. [86] developed a cell fate index algorithm from single-cell RNA-Seq data to assess reprogramming progression and found a slower progression of iCM reprogramming in human cells than that in mouse cells. Therefore, it is very important to develop an optimized protocol for the efficient reprogramming of human cells through a better understanding of its mechanism and eliminating more barriers. Indeed, an analytic technique, called trajectory alignment, identified barriers of MyoD-mediated human myogenic reprogramming, which could also be a helpful technique for identifying the barriers of human iCM reprogramming [111]. Furthermore, studying the safety and functional benefits of human iCM reprogramming with a large animal model (i.e., pig) is required for clinical translation of this technology; however, there is no successful report yet, although we have speculated that the *in vivo* environment of the heart should improve human cardiac reprogramming and yield fully functional iCMs in pig hearts [112]. Therefore, more effort should be invested in human cardiac reprogramming. A full understanding of direct cardiac reprogramming in human cells is a necessary requirement in order to consider its clinical applications. 

## 6. Conclusions

Direct *in vivo* cardiac reprogramming allows the direct injection of reprogramming cocktails into injured heart and *in situ* regeneration without cell transplantation and is a promising technology for the replacement of damaged myocardium in ischemic heart diseases. Although enormous progresses have been made recently, the technology of direct cardiac reprogramming is still under development for future clinical applications. Better mechanistic understanding is required, particularly for *in vivo* reprogramming, the possibility of reprogramming a chronic cardiac scar, and human iCM reprogramming, in order to translate it to a promising alternative to cell-based therapies. Nevertheless, we are optimistic that the rapid development of iCM reprogramming technology will offer a new therapeutic treatment for millions of patients with ischemic heart diseases in the future.

## Figures and Tables

**Table 1:** A summary of the mechanistic understanding of recent publications on mouse and human iCM reprogramming.

Signaling Pathways	Mouse iCM Reprogramming	Human iCM Reprogramming
TGFβ Inhibition	Enhanced by suppression of Smad2 and 3 phosphorylation [64,65]	Enhanced [60]
Akt1 Activation	Enhanced through activation of mTORC1 and Foxo3a in embryonic but not in adult fibroblasts [68]	Unknown
JAK inhibition	Enhanced iCM quality [19]	Unknown
RhoA-ROCK Inhibition	Enhanced through suppression of SRF-signaling [65]	Unknown
Notch Inhibition	Enhanced through increased Mef2c activity [67]	Unknown
Wnt Inhibition	Enhanced through suppression of canonical Wnt [60]	Enhanced [60]
FFV (Fgf2, FGF10, VEGF)	Enhanced through activation of p38 MAPK and PI3K/Akt pathways [69]	Unknown
**Inflammatory Signaling Pathways**		
Cyclooxygenase-2 Inhibition (by diclofenac)	Improved iCM quality through suppression of E2/PGE R4, cAMP/PKA, and IL1β [72]	Unknown
C-C chemokine inhibition	Enhanced by suppression of chemokine receptors [71]	Unknown
**Transcriptional Regulators**		
ZNF281 Activation	Enhanced by cooperation with Gata4 and suppression of inflammatory response [70]	Unknown
ZFPM2 Activation	Unknown	Enhanced [58]
**Epigenetic Factors**		
Bmi1 Inhibition	Improved induction of beating iCMs [80]	Unknown
Ezh2 Inhibition	Enhanced by suppression of H3K27me2 & H3K27me3 [78,79]	Unknown
G9a and GLP Inhibition	Enhanced by suppression of H3K9me & H3K9me2 [78]	Unknown
**Topological Factors**		
Microgroove ECM	Improved through nuclear localization of Mkl1 [91]	Unknown
3D Hydrogels	Improved by increased expression of MMPs [92]	Unknown
**Cell-cycle Manipulations**		
S-phase Synchronization	Accelerated by enhancing cell-cycle exit [66]	Unknown
G2/M-phase Synchronization	Improved iCM quality and yield [56]	Unknown
